# Oral Microbiome and Inferred Functions Predict Kaposi's Sarcoma Progression

**DOI:** 10.1002/jmv.70647

**Published:** 2025-10

**Authors:** Adam Officer, Wen Meng, David Spellman, Jeffrey Martin, Paige M. Bracci, Mike McGrath, Yufei Huang, Shou-Jiang Gao

**Affiliations:** 1 Cancer Virology Program, UPMC Hillman Cancer Center, Pittsburgh, Pennsylvania, USA; 2 Department of Microbiology and Molecular Genetics, University of Pittsburgh School of Medicine, Pittsburgh, Pennsylvania, USA; 3 Department of Epidemiology and Biostatistics, University of California San Francisco, San Francisco, California, USA; 4 Department of Medicine, The University of California at San Francisco, San Francisco, California, USA; 5 Department of Medicine, University of Pittsburgh School of Medicine, Pittsburgh, Pennsylvania, USA

**Keywords:** Cancer progression, inflammation, Kaposi′s sarcoma, KSHV lytic replication, microbiome, oral microbiome, short-chain fatty acids

## Abstract

Kaposi′s sarcoma (KS) is a common cancer among people living with HIV and is caused by infection with Kaposi′s sarcoma-associated herpesvirus (KSHV). While previous studies have linked the oral microbiome to KSHV infection and KS development, its role in KS progression remains poorly defined. We performed 16S rRNA gene sequencing targeting the V1-V2 and V3-V4 hypervariable regions to characterize the microbiome in 20 patients with AIDS-associated KS, including 10 with nonprogressive disease and 10 with progressive disease. Samples were obtained from three anatomical sites: oral cavity, peripheral blood, and tumor biopsies. The highest number of microbes were identified in the oral cavity at species, genus, and family levels. Beta diversity analysis revealed significant compositional differences in the oral microbiome between progressive and nonprogressive KS (*p* value = 0.044). Differential abundance analysis identified 16 species in the oral cavity associated with disease progression, compared to only three species in tumors and one in blood (*p* value < 0.05). Notably, *Prevotella pallens* and *Megasphaera micronuciformis*, both known producers of short-chain fatty acids, were significantly enriched in the oral microbiome of patients with progressive KS (log-fold changes = 2.8 and 2.4, respectively). Functional pathway inference revealed 39 differentially abundant microbial pathways in the oral cavity, including pathways related to denitrification, ubiquinone biosynthesis, and arginine metabolism (all *p* values < 0.05). Our findings provide the first evidence that specific oral microbiome alterations are associated with KS progression. The enrichment of short-chain fatty acid-producing bacteria and changes in microbial metabolic pathways may promote inflammation and KSHV lytic replication, offering potential mechanistic insights into KS pathogenesis and highlighting novel microbiome-based prognostic markers.

## 1 | Background

Kaposi′s sarcoma (KS) is a highly inflammatory and angiogenic malignancy driven by infection with Kaposi′s sarcoma-associated herpesvirus (KSHV), also known as human herpesvirus 8 [1]. KS arises predominantly in immunocompromised individuals, including persons living with HIV (PLWH, epidemic KS) and recipients of immunosuppressive therapy following organ transplantation (iatrogenic KS) [2]. However, KS also occurs in immunocompetent hosts, most notably among elderly men in Mediterranean and Eastern European regions (classical KS) and in endemic regions of sub-Saharan Africa (endemic KS). Early KS lesions appear as flat, irregularly shaped macules with subtle vascular proliferation and dilated, thin-walled vessels, often accompanied by sparse inflammatory infiltrates [2]. As the disease progresses, lesions develop into raised nodules that frequently ulcerate, exhibiting proliferative spindle-shaped cells forming slit-like vascular spaces filled with red blood cells [2].

KSHV, a gammaherpesvirus, has a biphasic lifecycle characterized by latent and lytic replication. Most KS tumor cells express the latent protein LANA (ORF73), and a small subset also expresses lytic proteins, particularly in early lesions, implicating both latent and lytic viral phases in KS pathogenesis [3]. Latency enables long-term persistence, immune evasion, and oncogenic transformation, whereas lytic replication produces infectious virions and viral proteins that exacerbate inflammation, angiogenesis, and immune activation [4]. Pharmacologic inhibition of herpesvirus lytic replication has shown promise in preventing KS development in certain patient cohorts [5].

Inflammation is a hallmark of KS and plays a central role in its pathogenesis [6]. Chronic inflammation fosters angiogenesis, immune suppression, and tumor proliferation, creating a permissive microenvironment for tumor growth [7, 8]. Oxidative stress and inflammatory cytokines such as IL-6, TNF-α, and IL-18, produced in response to both latent and lytic KSHV infection, amplify this inflammatory milieu and can reactivate latent KSHV [9-11]. KSHV *de novo* infection and viral products, including vIL-6, vGPCR, vIRFs, and miRNAs, further induce inflammatory and angiogenic responses, reinforcing a feed-forward loop of inflammation, viral replication, and tumor cell expansion [3, 12-18].

Additional sources of inflammation contribute to KS pathobiology. In PLWH, persistent immune activation and chronic inflammation arise from viral replication, bacterial coinfections, and HIV-induced dysregulated metabolism [19, 20]. Iatrogenic KS is linked to immune dysregulation from immunosuppressive drugs, whereas classical KS is associated with aging-related immune decline and oxidative stress [2]. In endemic KS, coinfections and chronic skin exposure to environmental agents such as iron may exacerbate inflammation and oxidative damage [21].

A growing body of evidence highlights the role of the microbiome, particularly bacterial communities, in modulating inflammation and KSHV pathogenesis [20]. Certain bacteria produce short-chain fatty acids (SCFAs) such as butyrate and propionate, which can induce KSHV lytic reactivation and inflammation [22-24]. These SCFAs are abundant in the oral cavity and gastrointestinal tract, sometimes reaching millimolar concentrations in saliva, well above the thresholds required to trigger KSHV lytic gene expression in vitro [24]. Furthermore, KSHV-encoded miRNAs can activate the alternative complement pathway and sensitize cells to bacterial pathogen-associated molecular patterns (PAMPs) via Toll-like receptors (TLRs), enhancing the inflammatory response to bacterial components such as lipopolysaccharide (LPS), flagellin, and lipoteichoic acid (LTA) [9, 25, 26]. Mechanistic studies have shown that *Staphylococcus aureus* can induce KSHV lytic replication via TLR2 [27], while *Porphyromonas gingivalis* and *Pseudomonas aeruginosa* flagellin promote cellular proliferation through TLR4- and TLR5-mediated activation of ERK1/2, p38 and JNK signaling [26, 28].

Importantly, next-generation sequencing studies have revealed that the oral microbiome of patients with oral KS differs significantly from that of matched controls without oral KS, suggesting a potential link between microbial composition and KS pathogenesis [29]. These findings underscore the potential of the microbiome to influence KSHV infection dynamics and the inflammatory tumor microenvironment.

While the incidence of KS has decreased dramatically with the advent of antiretroviral therapy (ART) for HIV infection in resource-rich setting, KS incidence [30] and KS mortality [31] remain high in other parts of the world, particularly sub-Saharan Africa. The high burden of KS in this setting is attributed to the widespread prevalence of both KSHV and HIV, compounded by limited access to effective health care, specifically HIV treatment and KS-specific care [32]. ART has been shown to reduce both KSHV load and the incidence of KS in PLWH, but further understanding of microbial cofactors may reveal additional therapeutic and preventive strategies [32], which is especially important for patients for whom ART is not effective.

In this study, we investigated the bacterial microbiome in patients from sub-Saharan Africa with biopsy-confirmed KS [33]. We compared patients with progressive versus nonprogressive disease and analyzed microbiome profiles from three body sites: the oral cavity, peripheral blood, and tumor tissue. Although alpha diversity did not significantly differ between disease groups in any sample type, beta diversity differences were most pronounced in the oral cavity. Specific bacterial species, genera, and families were enriched in patients with progressive disease and correspond with previously identified mechanistic links to inflammation and KSHV reactivation. These findings provide new insights into the microbiome′s role in KS progression and could serve as the basis for the development of microbiome-targeted strategies for prevention and treatment.

## 2 | Materials and Methods

### 2.1 | Study Cohort

This is a retrospective study using repository specimens and data deposited to the AIDS and Cancer Specimen Resource (ACSR). The University of Pittsburgh Institutional Review Board (IRB) determined that the study is not research involving human subjects as defined by DHHS and FDA regulations and waived of ethical oversight (STUDY23090059). Human Ethics and Consent to Participate declarations is not applicable for this study.

Unstimulated saliva, KS tissue, and peripheral blood specimens collected at baseline before treatment from participants enrolled in the Antiretrovirals for Kaposi′s Sarcoma (ARKS) cohort were accessed through ACSR. The ACSR provided limited, unlinked, deidentified demographic and clinical study metadata for the 20 randomly selected cases that were analyzed: 10 patients with KS progression defined as requiring chemotherapy for their KS or death before study end (progressors) and 10 patients without KS progression (non-progressors).

The ARKS cohort recruited participants with AIDS-KS in Kampala, Uganda, and followed them longitudinally for 1 year to evaluate their response to ART [34]. Eligibility criteria included histologically-confirmed KS via skin punch biopsy and no prior history of ART use. Individuals with advanced KS defined as requiring urgent chemotherapy or having functionally disabling KS-related complications were excluded [34]. Additional exclusion criteria included evidence of untreated active opportunistic infections (e.g., tuberculosis, cryptococcosis) or concurrent malignancies.

### 2.2 | DNA Extraction, 16S Amplification, and Library Preparation

Genomic DNA was extracted from saliva and PBMC pellets using the Qiagen DNeasy Blood & Tissue Kit (Cat #51306) according to the manufacturer′s protocol, with minor modifications to the cell lysis step. Briefly, 200 μL of saliva or PBMC sample was centrifuged at 5,000 × *g* for 10 min to obtain pellet. The pellet was resuspended in 180 μL of 20 mg/mL lysozyme and incubated at 37°C for 30 min. Subsequently, 20 μL of proteinase K and 200 μL of Buffer AL were added, followed by incubation at 56°C for 30 min and an additional incubation at 95°C for 15 min. DNA was then purified per the manufacturer′s protocol. Tumor tissue was preserved with RNAlater and DNA was extracted as previously described [35].

The bacterial 16S rRNA gene was amplified using two primer sets targeting different hypervariable regions. The V1-V2 region was amplified with forward primer 5′-AGAGTTTGATCMTGGCTCAG-3′ and reverse primer 5′-CTGCCTCCCGTAGGAGT-3′. The V3-V4 region was amplified using forward primer 5′-CCTACGGGNGGCWGCAG-3′ and reverse primer 5′-ACTACHVGGGTATCTAATCC-3′. Amplicons were pooled and sequenced on an Illumina NovaSeq. 6000 platform following the manufacturer′s guidelines.

### 2.3 | Bioinformatic Processing

Raw sequence data were processed using the nf-core/ampliseq v2.11.0 workflow. Initial trimming was performed using cutadapt to remove 16S primers and terminal poly-G artifacts. Quality control and sequence variant inference was performed using DADA2. This included removal of PhiX contamination, trimming of low-quality bases (median quality score threshold of 25 and minimum length 279 bp), and exclusion of reads with more than two expected errors. Paired-end reads were merged, and PCR chimeras were removed.

The final data set yielded 19,854 unique amplicon sequence variants (ASVs) across all samples. On average, 52% of raw reads per sample were retained after filtering, ranging from 35% to 75%. The final ASV table consisted of 23,301,638 total reads, with individual sample counts ranging from 10,240 to 1,431,772 (mean: 388,361). Taxonomic classification was performed using the Silva SSU rRNA database (release 138.1). ASVs with confidence scores less than 0.8 or classified as mitochondrial or chloroplast sequences were excluded.

A microbe was considered “detected” in an individual sample if it met both of the following criteria: (1) > 5 assigned reads and (2) a relative abundance > 1% of total reads in that sample. A microbe was considered “detected” in a specific sample type (oral, blood, or tumor) if it was detected in ≥ 25% of all samples of that type, irrespective of disease status.

### 2.4 | Statistical Analysis

Clinical features between progressive and nonprogressive KS cases were compared using Welch's t-test (“t-test” in the text for simplicity) implemented in scipy (v1.14.1) using Python (v3.12.8).

Microbiome statistical analyses were conducted in R (v4.4.2) using the phyloseq (v1.50.0) [36] and vegan (v2.6.10) packages. Chao1 and Shannon alpha diversity indices were computed on rarefied ASV tables using the estimate_richness function in phyloseq. Weighted Bray-Curtis dissimilarity matrices were generated from rarefied ASV tables, and beta diversity was analyzed via principal coordinate analysis (PCoA) using the cmdscale function. Significance of group separation was tested using permutational multivariate analysis of variance (PERMANOVA), as implemented in the adonis2 function from the vegan package, with 10,000 permutations [37]. Rarefaction, where relevant, was performed to an even depth of 9000 reads per sample.

Differential abundance of microbes was assessed using ALDEx2 [38] with 128 Monte Carlo instances and the Wilcoxon rank-sum test. Functional pathway inference was performed with PICRUSt2 (v2.3.0), and differences in predicted pathway abundances between groups were tested using the Wilcoxon rank-sum test. All tests were two-sided, and a nominal p value of less than 0.05 was considered statistically significant for all analyses.

## 3 | Results

### 3.1 | Clinical Features of Patients by Disease Status

We analyzed microbiome profiles and clinical characteristics from 20 patients, comprising 10 individuals with progressive KS and 10 with nonprogressive disease. Of 17 demographic and clinical variables, 16 showed no statistically significant differences between the two groups (*p* value > 0.05; Table 1). However, blood d-dimer levels were modestly elevated in patients with progressive disease compared to those with nonprogressive disease (*p* value = 0.035; Table 1). No significant differences were observed in HIV viral load (*p* value = 0.913) or CD4 + T cell counts (*p* value = 0.629) between the two groups. KSHV viral DNA was detected in blood in 6 of the 20 patients, with no significant difference in viral load between progressors and non-progressors (*p* value = 0.111). KSHV serology, as measured by antibody levels to KSHV ORF-K8.1 and ORF65 proteins, were also not significantly different between progressors and non-progressors (*p* value = 0.691 for ORF-K8.1 and *p* value = 0.075 for ORF65).

### 3.2 | Diversity of Microbiome From Different Anatomical Sites

**FIGURE 1 |:**
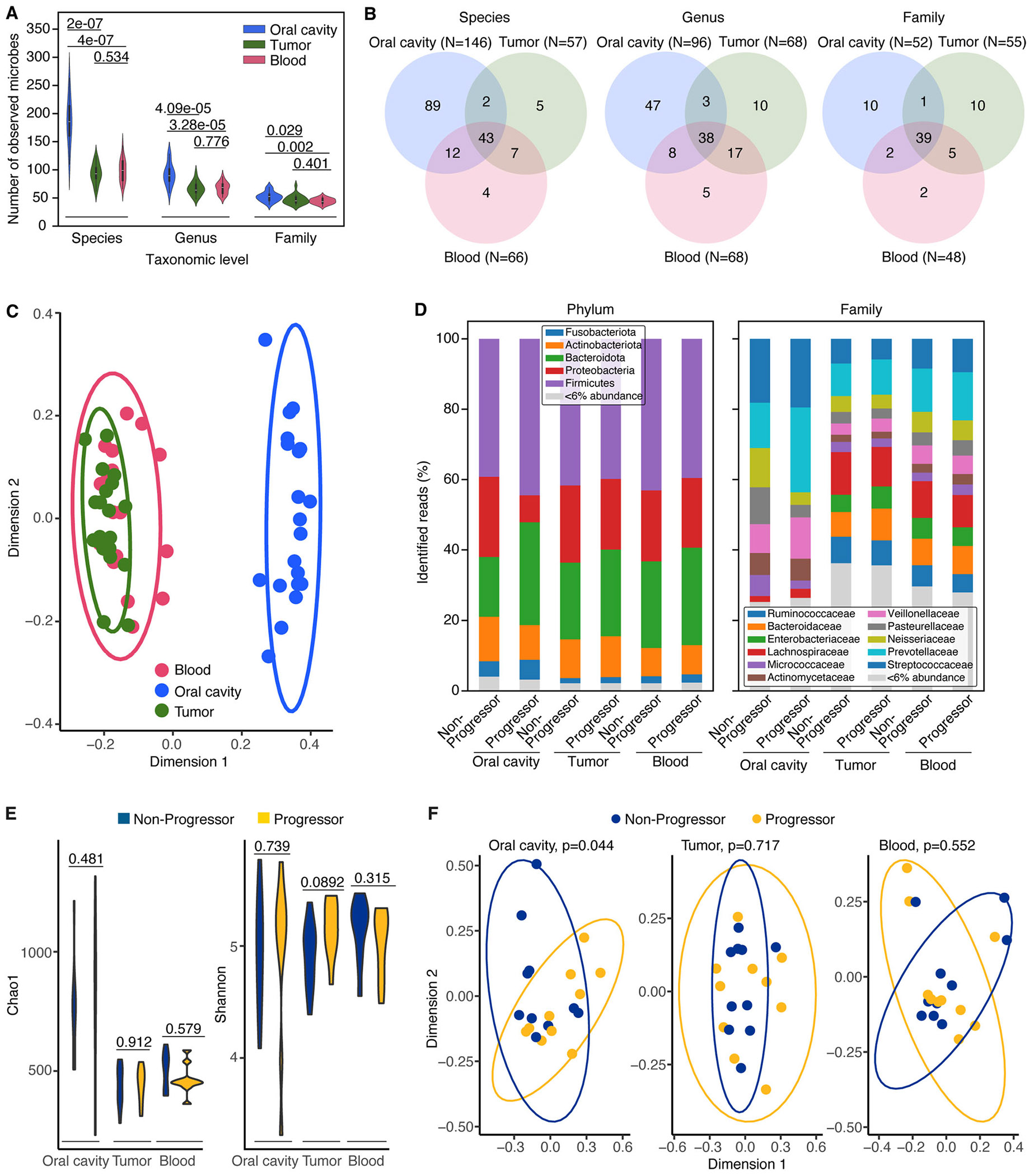
Global microbiome alterations in progressive Kaposi′s sarcoma (KS). (A) Numbers of observed microbes across sample types (oral cavity, tumor and blood) at species, genus, and family taxonomic levels. (B) Overlap of observed microbes among anatomical sites at species, genus, and family taxonomic levels, highlighting greater similarity between tumor and blood microbiomes compared to the oral cavity. (C) Principal coordinates analysis (PCoA) based on Bray-Curtis distances showing distinct separation of oral microbiomes from tumor and blood samples (PERMANOVA, *p* value < 0.001). (D) Phylum- and Family-level relative abundance profiles reveal distinct microbial community patterns with progressive or nonprogressive KS in the oral cavity compared to tumor and blood. (E) Alpha diversity assessed by Shannon and Chao1 indices shows no significant differences between progressive and nonprogressive KS across all sample types. (F) PCoA plots stratified by sample type reveal global microbiome composition differences by disease status only in the oral cavity, with no significant separation observed in tumor or blood microbiomes.

Microbial communities were profiled from three distinct anatomical compartments, including oral cavity, peripheral blood, and tumor tissue, using 16S rRNA gene sequencing targeting the V1-V2 and V3-V4 hypervariable regions (see Materials and Methods). Among all sample types, oral samples had the highest numbers of observed taxa across all taxonomic levels. Significantly greater numbers of observed taxa at the species (*p* value = 2.2 ×10^−7^ and 4.2 ×10^−7^), genus (*p* value = 4.1 ×10^−5^ and 3.3 ×10^−5^), and family levels (*p* value = 0.029 and 0.002) were identified in oral samples compared to blood and tumor samples (Figure 1A). There were no significant differences in the number of observed taxa between tumor and blood samples across these three taxonomic levels (*p* value = 0.534, 0.776 and 0.401 for species, genus, family, respectively)

Analysis of taxonomic overlap revealed that the proportion of shared microbial taxa across the three anatomical sites was consistent across taxonomic levels (Figure 1B). However, tumor samples shared more microbes with blood than with the oral cavity, suggesting potential microbial translocation from circulation into KS lesions. For instance, at the genus level, 55 of the 68 genera identified in tumor samples overlapped with those found in blood, whereas only 41 overlapped with oral samples (Mantel-Haenszel chi square, value 6.891, *p* value < 0.01) despite a higher total number of genera identified in oral samples compared to blood (96 vs. 68, respectively; Figure 1B). This pattern supports the hypothesis that KS tumors, due to their leaky vasculature, may be more influenced by blood-borne microbes than by microbes originating from the oral cavity.

Global microbial composition varied significantly by anatomical site. The oral microbiome was distinct from both blood and tumor microbiomes (*p* value < 0.0001; Figure 1C), while microbial profiles from blood and tumor samples were not significantly different from one another (*p* value = 0.608; Figure 1C). This finding reinforces the hypothesis that KS tumors, due to their leaky vasculature, may be colonized by blood-borne microbes.

Across all sample types, five bacterial phyla and eleven families were present with a relative abundance > 6% (Figure 1D). The oral cavity microbiome was dominated by members of the phylum *Firmicutes*, and by the families *Streptococcaceae*, *Prevotellaceae*, and *Pasteurellaceae*. In contrast, both blood and tumor microbiomes were enriched for *Firmicutes* and *Proteobacteria*, with *Prevotellaceae*, *Lachnospiraceae* and *Bacteroidaceae* being the most consistently represented families in both compartments (Figure 1D).

When stratifying by disease status, global microbial composition within the blood and tumor compartments did not significantly differ between progressive and nonprogressive disease groups (*p* value = 0.552 and 0.717, respectively; Figure 1E). However, microbiome composition in the oral cavity showed a significant difference by disease status (*p* value = 0.044; Figure 1E), suggesting that the oral microbiome may reflect or influence KS disease progression.

Microbial alpha diversity, measured by both Shannon and Chao1 indices, did not differ significantly between progressive and nonprogressive disease groups in any of the three anatomical sites (Shannon: *p* value = 0.481, 0.912 and 0.579; and Chao1: *p* value = 0.739, 0.089 and 0.315 for oral cavity, tumor, and blood, respectively; Figure 1F), indicating that the observed compositional shifts are not driven by changes in overall microbiome richness or evenness.

### 3.3 | Association of Specific Microbes With Disease Status

**FIGURE 2 |:**
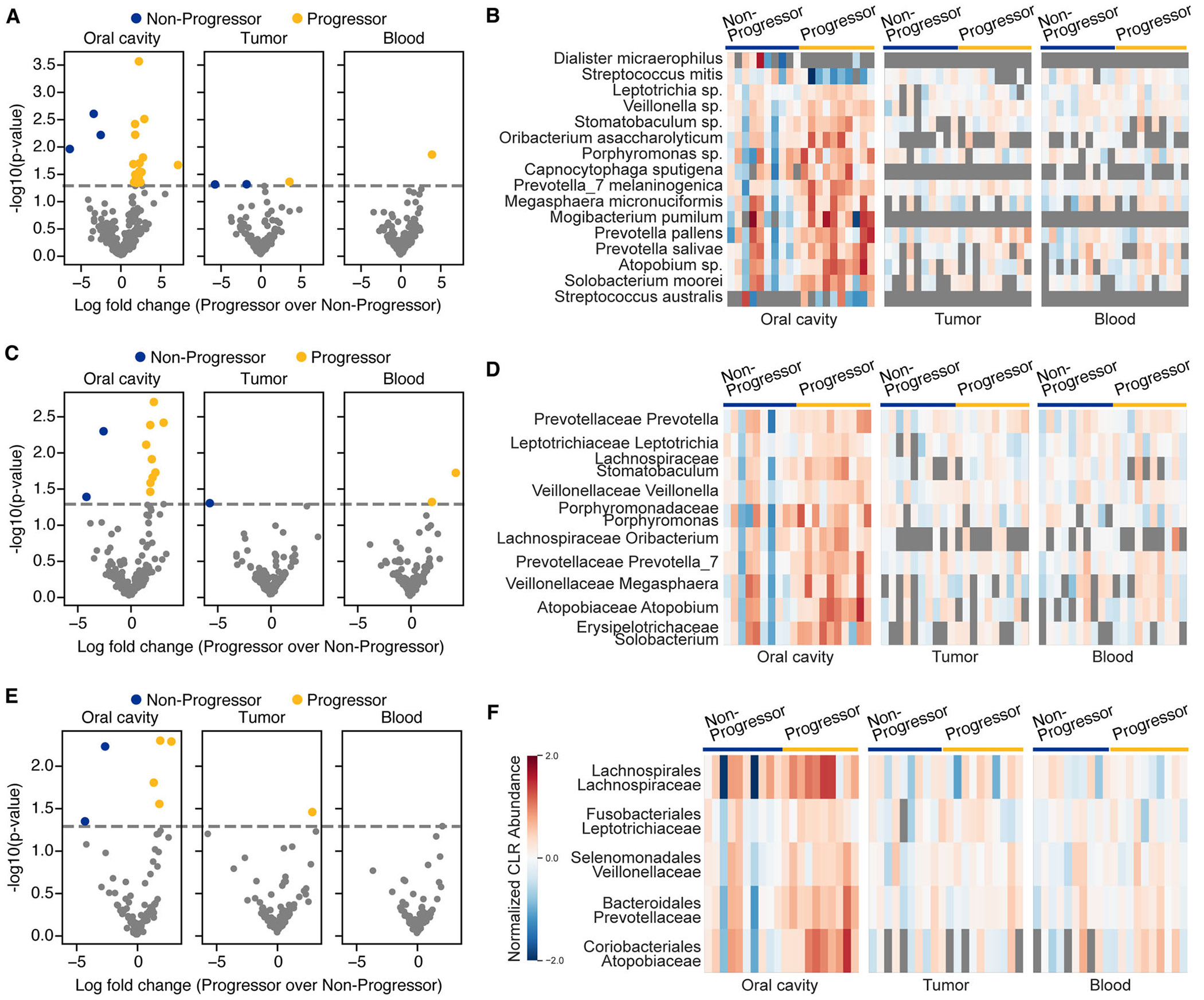
Disease progression-associated differential microbes across anatomical sites. (A, C, E) Volcano plots showing differential abundance of microbial taxa at (A) species, (C) genus, and (E) family levels in progressive versus nonprogressive Kaposi′s sarcoma. (B, D, F) Heatmaps depicting relative abundance of significantly differential taxa across sample types at (B) species, (D) genus, and (F) family levels (ALDEx2, *p* value < 0.05).

Differential abundance analysis revealed that the oral cavity harbored the greatest number of microbial species associated with KS disease progression. Specifically, 16 species in the oral microbiome were significantly associated with disease status, compared to only three species in tumor samples and one species in blood (*p* value < 0.05; Figure 2A). Among the oral microbes enriched in patients with progressive KS were *Prevotella pallens* and *Megasphaera micronuciformis*, showing log-fold changes (LFC) of 2.8 and 2.4, respectively (Figure 2B). Additional species associated with progressive disease included *Streptococcus australis, Mogibacterium pumilum, Prevotella salivae, Solobacterium moorei*, and *Capnocytophaga sputigena* (Figure 2B). In contrast, only two species, *Streptococcus mitis* and *Dialister micraerophilus*, were found to be enriched in patients with nonprogressive KS (Figure 2B).

At the genus level, 10 genera were differentially abundant in the oral cavity, compared to only one in the tumor and two in the blood (*p* value < 0.05; Figure 2C). Genera enriched in progressive disease included *Prevotella, Prevotella_7, Veillonella, Atopobium, Stomatobaculum*, and *Megasphaera* (Figure 2D). A similar pattern emerged at the family level: five differentially abundant families were identified in the oral cavity, one in the tumor, and none in the blood (*p* value < 0.05; Figure 2E and F).

**FIGURE 3 |:**
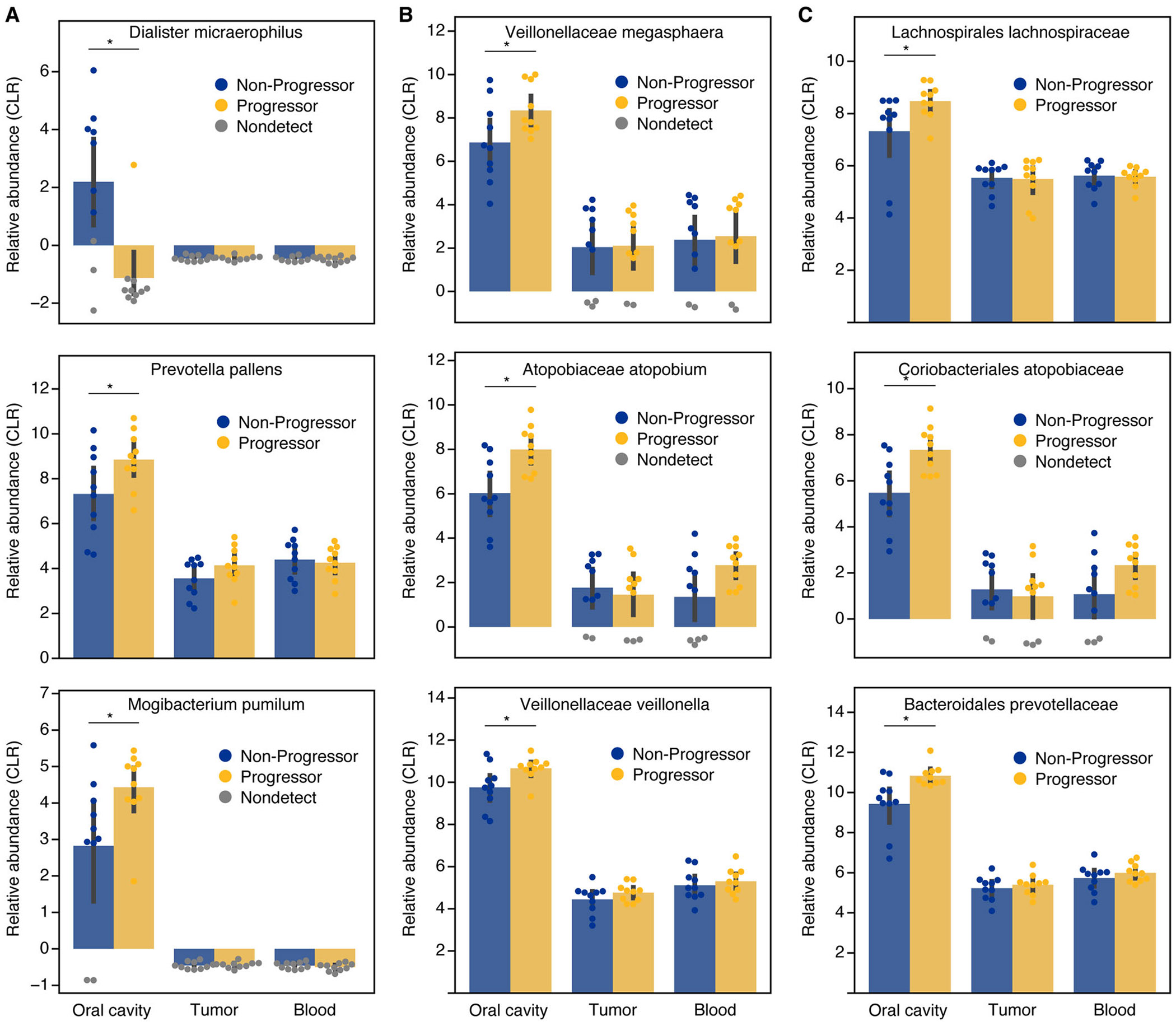
Specific microbial taxa enriched or depleted in the oral cavity of progressive Kaposi′s sarcoma. (A, B, C) Relative abundance of selected differential microbes identified in all sample types at the (A) species, (B) genus, and (C) family levels, highlighting taxa enriched in progressive disease.

Key differentially abundant taxa across multiple taxonomic levels, including *Dialister micraerophilus, Megasphaera* (family *Veillonellaceae*), and *Veillonella*, are highlighted in Figure 3. These findings further support the hypothesis that the oral microbiome is more closely associated with KS disease progression than the microbiota of blood or tumor tissues.

### 3.4 | Taxonomic Relationships of Oral Microbes Associated With Disease Status

**FIGURE 4 |:**
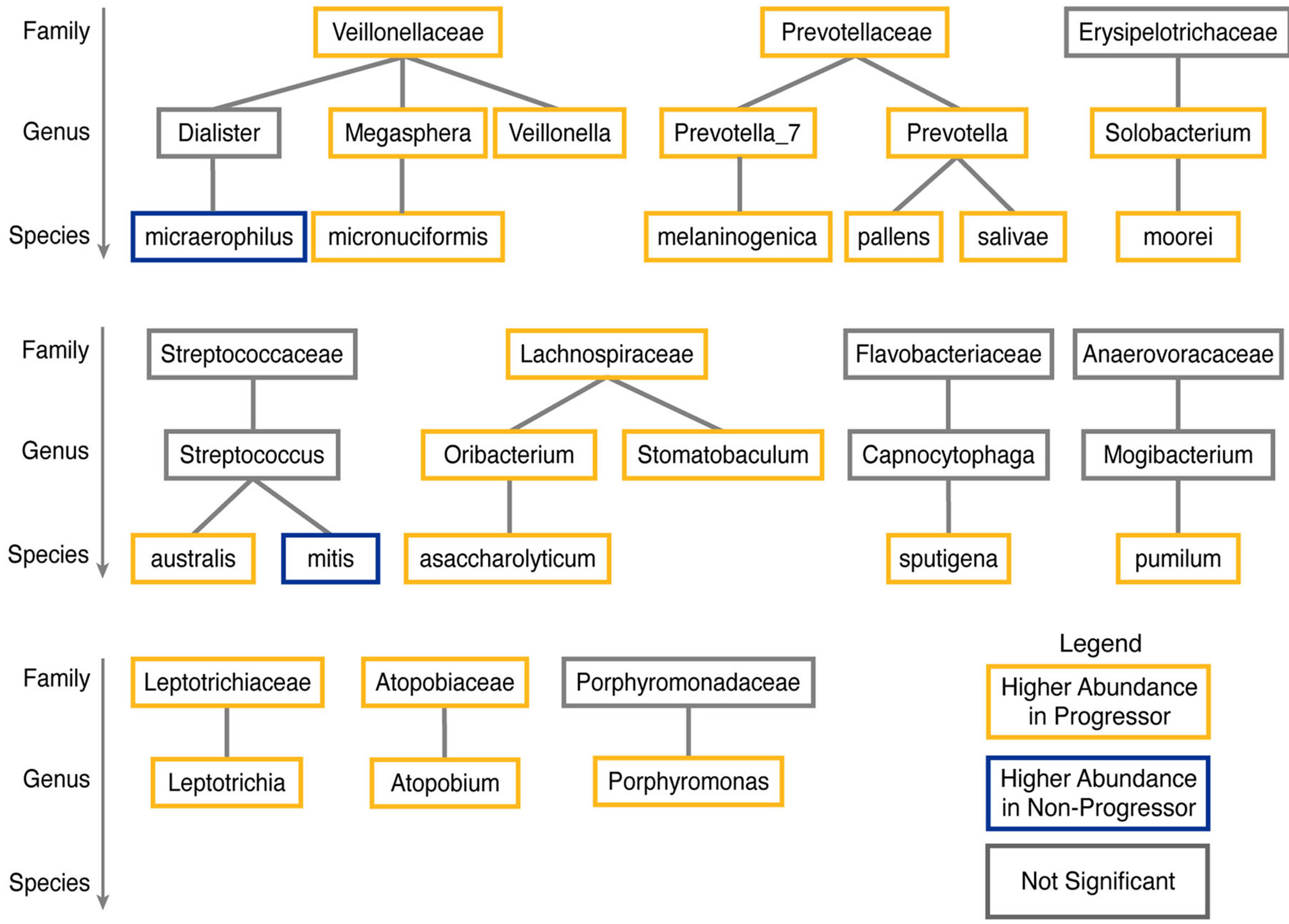
Phylogenetic structure of differential microbes associated with progressive Kaposi′s sarcoma (KS) in the oral microbiome. Taxonomic tree depicting the hierarchical relationships among differentially abundant microbial species, genera, and families enriched or depleted in the oral cavity of patients with progressive KS.

Many of the differentially abundant microbes identified in the oral cavity were taxonomically related, suggesting a hierarchical organization of microbial shifts associated with disease progression. At the genus level, six of the nine species found to be differentially abundant were matched by corresponding genus-level changes, indicating consistency across taxonomic scales (Figure 4). At higher taxonomic ranks, members of the family *Prevotellaceae* were consistently enriched in patients with progressive disease. Within this family, both *Prevotella* and *Prevotella*_7 were significantly more abundant in progressive cases, as were three constituent species: *Prevotella melaninogenica, Prevotella pallens*, and *Prevotella salivae*. Similarly, *Megasphaera micronuciformis* (from the genus *Megasphaera*) and the genus *Veillonella* were also more abundant in progressive disease. Both genera belong to the *Veillonellaceae* family, which itself was significantly enriched in patients with progressive disease. These findings underscore taxonomically structured microbial shifts that may contribute to, or result from, KS progression.

### 3.5 | Functional Inference of Oral Microbes Associated With Disease Status

**FIGURE 5 |:**
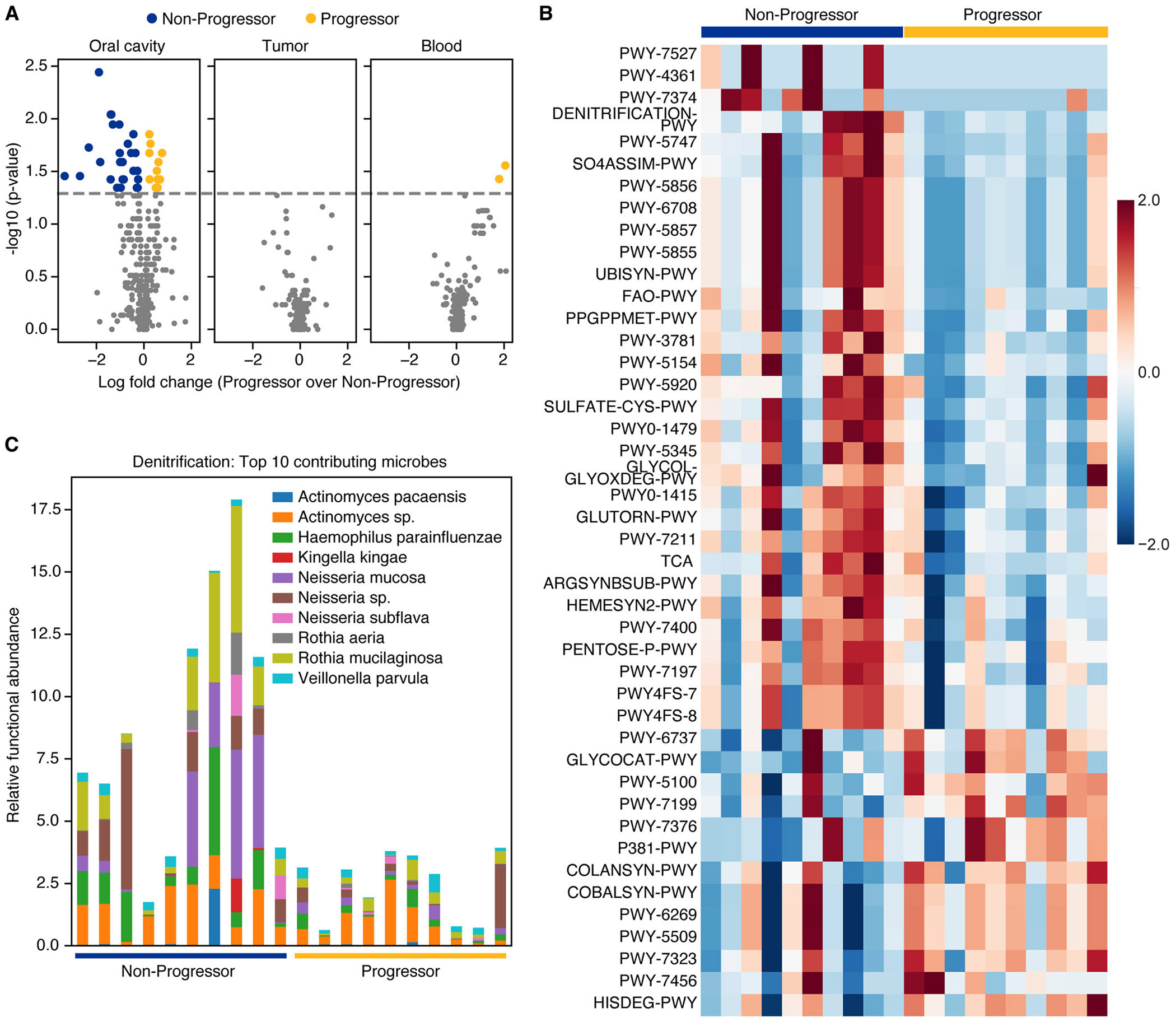
Inferred microbial functional alterations associated with Kaposi′s sarcoma progression. (A) Volcano plots of PICRUSt2-inferred pathway activity comparing progressive to nonprogressive disease across the oral cavity, tumor, and blood microbiomes. (B) Differential microbial pathways identified in the oral cavity, with emphasis on pathways related to denitrification, ubiquinone biosynthesis, and arginine metabolism. (C) Microbe-level loadings showing higher relative levels of microbes capable of denitrificiation observed in nonprogressive disease.

Functional inference using PICRUSt2 revealed that differentially enriched microbial metabolic pathways were exclusively observed in the oral microbiome, with no significant pathway-level differences detected in blood or tumor samples (Figure 5A). Interestingly, most upregulated pathways were associated with nonprogressive disease, whereas relatively few pathways showed increased activity in progressive KS. Notably, several pathways enriched in nonprogressive disease were involved in coenzyme Q and ubiquinone biosynthesis (*PWY-7374, PWY-5856, PWY-6708, PWY-5857, PWY-5855, UBISYN-PWY*) and denitrification (*DENITRIFICATION-PWY*) (Figure 5B). The inferred activity of the denitrification pathway was strongly associated with the presence of *Neisseria* and *Rothia* genera in the oral cavity (Figure 5C), suggesting that specific oral commensals may confer functional properties linked to disease stabilization.

## 4 | Discussion

In this study, we investigated for the first time the associations between the microbiome and disease progression in patients with AIDS-KS using multi-site sampling and 16S rRNA gene sequencing. We observed minimal differences in baseline clinical characteristics between patients with progressive versus nonprogressive KS, except for elevated d-dimer levels in progressive disease. Elevated d-dimer, a protein fragment released when blood clots break down, has been previously associated with increased mortality risk in HIV-positive individuals [39] and is correlated with HIV viral load [40], suggesting that disease progression in KS may reflect broader markers of systemic inflammation and HIV disease burden. Although a prior study identified C-reactive protein (CRP) as a serum biomarker of progressive KS [41], CRP was elevated but not significantly different in our cohort, likely due to being statistically underpowered. Additionally, consistent with prior reports, KSHV DNA was detectable in only a fraction of peripheral blood samples, with no significant difference in viral load between disease groups [42-45], highlighting the limitations of peripheral KSHV DNA as a prognostic biomarker.

We profiled the microbiome in three anatomical compartments including oral cavity, peripheral blood, and KS tumors. Consistent with a prior study [46], the oral cavity harbored significantly higher microbial richness across all taxonomic levels compared to blood and tumor samples. Our analyses confirmed that the oral microbiome was compositionally distinct from both blood and KS tumor microbiomes, whereas the latter two were not significantly different from each other. This observation supports the hypothesis that the leaky vasculature characteristic of KS tumors may facilitate microbial translocation from the bloodstream into tumor lesion, resulting in overlapping microbial profiles.

At the phylum and family levels, our results aligned with previously characterized microbial landscapes in healthy donors. The oral cavity was dominated by *Pasteurellaceae, Streptococcaceae*, and *Prevotellaceae*, three families that are known to be dominant in the oral cavity of healthy donors [46], whereas blood and tumor samples had high relative abundance of the phyla *Proteobacteria* and *Firmicutes* [47, 48]. This finding is consistent with prior studies on the microbiome of PLWH, which also observed high relative abundance of these two phyla in the serum and the oral cavity [49, 50]. This consistency reinforces the validity of our microbiome profiling and suggests that the underlying microbial community structures in KS are not globally disrupted by disease progression.

Despite the absence of significant changes in overall microbial richness (alpha diversity) or global composition (beta diversity) in tumor or blood samples, we identified significant progression-associated changes in the oral microbiome. Although alpha diversity (Shannon and Chao1 indices) was not significantly altered by disease status, beta diversity (PCoA using the Weighted Bray-Curtis distance) in the oral cavity was significantly different between progressive and nonprogressive disease. These findings echo prior studies in melanoma [51], cervical cancer [52] and oral squamous carcinoma [52], where microbiome diversity metrics have been linked to clinical outcomes. The lack of diversity differences in KS tumors may be due to the unique immune environment of HIV-infected individuals or limited statistical power.

Differential abundance analysis using ALDEx2 identified numerous microbial taxa in the oral cavity associated with disease status. Specifically, the oral microbiome exhibited far more differential taxa than either blood or tumor microbiomes. This finding is particularly relevant given that 90% of patients (18 of 20) in our cohort presented with oral KS lesions, suggesting that the oral microbiome may directly influence the local tumor microenvironment.

Two species, *Prevotella pallens* and *Megasphaera micronuciformis*, were significantly more abundant in progressive KS and are known producers of SCFAs, such as butyrate. SCFAs have been shown to induce KSHV reactivation in vitro [22]. The oral cavity of healthy individuals contains up to 30 mM of butyrate [24], and an increased abundance of SCFA-producing microbes may locally amplify this concentration, promoting KSHV reactivation and KS progression. Additional enriched genera in progressive KS included *Megasphaera* and *Veillonella*, both of which produce LPS and flagellin, further contributing to immune activation and KSHV lytic replication. These findings are consistent with prior studies in lung cancer, where higher levels of *Megasphaera* were associated with poor response to chemotherapy [53] and increased *Veillonella* abundance was observed in lung cancer patients relative to healthy controls [54].

In contrast, *Dialister micraerophilus* was enriched in nonprogressive disease. Interestingly, members of the *Dialister* genus have been previously observed at higher levels in both tumor and gut microbiomes of cancer patients compared to healthy controls [55, 56], including in KS oral lesions [29], suggesting a possible dual role in early tumorigenesis and later disease stabilization.

At the family level, *Lachnospiraceae, Atopobiaceae*, and *Prevotellaceae* were significantly enriched in progressive disease. Notably, *Atopobiaceae* has been associated with more aggressive HPV-positive cervical cancer [57], and *Prevotella* species have been repeatedly implicated in colorectal cancer pathogenesis [58], underscoring potential shared mechanisms of microbiome-driven tumor progression across cancer types.

Importantly, many of the differentially abundant taxa exhibited phylogenetic clustering. For instance, *Prevotella pallens, Prevotella salivae*, and *Prevotella melaninogenica*, all of which are enriched in progressive KS, are nested within the *Prevotella* genus and *Prevotellaceae* family, suggesting a coherent expansion of functionally similar microbial groups. Likewise, *Megasphaera* and *Veillonella*, both within *Veillonellaceae*, were also elevated in progressive disease. These findings suggest that disease-associated microbial shifts occur in a taxonomically and functionally coordinated manner, potentially driven by shared traits such as SCFA production, LPS and flagellin expression, and pro-inflammatory capacity.

Differential pathway activity was exclusively observed in the oral microbiome. Notably, pathways involved in coenzyme Q (ubiquinone) biosynthesis and denitrification were enriched in patients with nonprogressive disease. Microbial denitrification produces nitric oxide, a signaling molecule known to induce vasodilation and inhibit platelet adhesion [59], and may modulate endothelial permeability and angiogenesis in KS tumors. Nitric oxide has also been shown to promote KSHV reactivation in vitro [60]. Interestingly, the inferred denitrification pathway was strongly associated with the presence of *Neisseria* and *Rothia*, suggesting a possible protective role for these genera.

In summary, this study provides the first observational evidence linking oral microbiome composition and function to clinical outcomes in KS. Our findings highlight a potential role for SCFA-producing, pro-inflammatory bacteria in driving KSHV reactivation, inflammation and tumor progression, while suggesting that denitrifying and coenzyme Q-producing microbes may be associated with stable disease. These findings require experimental confirmation but lay the foundation for future longitudinal and interventional studies to validate microbiome-based biomarkers and explore therapeutic manipulation of the oral microbiome in KS management.

## Figures and Tables

**TABLE 1 |:** Comparison of clinical features of randomly selected ARKS study participants by disease status.

	Non-progressor (*N* = 10)	Progressor (*N* = 10)	*p* value
Log_10_ KSHV viral load (copies/mL)	1.9 (0.1)	2.4 (1.1)	0.111
Log_10_ HIV viral load (copies/mL)	5.3 (0.5)	5.3 (0.4)	0.913
K8.1 Serology density (OD)	2.2 (2.2)	1.8 (2.2)	0.691
ORF65 Serology density (OD)	3.2 (2.6)	1.3 (1.6)	0.075
CD4 Count (cells/mm^3^)	102 (113)	134 (177)	0.629
Age (years)	31.2 (5.7)	34 (11)	0.479
CRP (μg/mL)	9.8 (11.9)	19.4 (24.2)	0.275
HMGB1 (ng/mL)	5.2 (1.7)	6.1 (3.0)	0.424
IP10 (pg/mL)	630 (447)	665 (558)	0.877
d-dimer (μg/mL)	1.3 (0.5)	3.3 (2.7)	**0.035** *
sCD14 (ng/mL)	2342 (884)	2220 (761)	0.746
sCD27 (U/mL)	352 (154)	653 (593)	0.137
sCD163 (ng/mL)	1660 (1271)	2508 (1901)	0.257
Trp (ng/mL)	4213 (1298)	4597 (2421)	0.664
Kyn (ng/mL)	519 (166)	570 (190)	0.535
Kyn/Trp ratio	0.12 (0.03)	0.16 (0.11)	0.382
TNFR1 (pg/mL)	1734 (615)	2065 (894)	0.348

* Mean (standard deviation) are shown; *OD* optical density; *p* value from Welch's t-test.

## Data Availability

The data that support the findings of this study are available on request from the corresponding author. The data are not publicly available due to privacy or ethical restrictions. The datasets generated during the study are available at the Sequence Read Archive with accession number: PRJNA1297116 and are currently set to be publicly released on December 31, 2025 or once the manuscript is accepted. The Editors and reviewers can assess the datasets at: https://dataview.ncbi.nlm.nih.gov/object/PRJNA1297116?reviewer=ld0qtm0lrjd78qioru1rifkbjo All code to reproduce these analyses is available on GitHub at https://github.com/adofficer/ks_microbiome.
